# AbeTx1 Is a Novel Sea Anemone Toxin with a Dual Mechanism of Action on Shaker-Type K^+^ Channels Activation

**DOI:** 10.3390/md16100360

**Published:** 2018-10-01

**Authors:** Diego J. B. Orts, Steve Peigneur, Laíz Costa Silva-Gonçalves, Manoel Arcisio-Miranda, José Eduardo P. W. Bicudo, Jan Tytgat

**Affiliations:** 1 Department of Physiology, Institute of Biosciences, University of São Paulo, 05508-090 São Paulo, Brazil; diego.orts@usp.br (D.J.B.O.); jebicudo@usp.br (J.E.P.W.B.); 2 Toxicology and Pharmacology, University of Leuven (KU Leuven), Campus Gasthuisberg O&N2, Herestraat 49, P.O. Box 922, 3000 Leuven, Belgium; steve.peigneur@pharm.kuleuven.be; 3 Laboratório de Neurobiologia Estrutural e Funcional (LaNEF), Departamento de Biofísica, Universidade Federal de São Paulo, 04023-062 São Paulo, Brazil; laiz.lcs@gmail.com (L.C.S.-G.); arcisio.miranda@unifesp.br (M.A.-M.)

**Keywords:** sea anemone neurotoxin, *Actinia bermudensis*, potassium channel, type 6 K_V_-toxins, Alanine point mutation

## Abstract

Voltage-gated potassium (K_V_) channels regulate diverse physiological processes and are an important target for developing novel therapeutic approaches. Sea anemone (Cnidaria, Anthozoa) venoms comprise a highly complex mixture of peptide toxins with diverse and selective pharmacology on K_V_ channels. From the nematocysts of the sea anemone *Actinia bermudensis*, a peptide that we named AbeTx1 was purified and functionally characterized on 12 different subtypes of K_V_ channels (K_V_1.1–K_V_1.6; K_V_2.1; K_V_3.1; K_V_4.2; K_V_4.3; K_V_11.1; and, Shaker IR), and three voltage-gated sodium channel isoforms (Na_V_1.2, Na_V_1.4, and BgNa_V_). AbeTx1 was selective for Shaker-related K^+^ channels and is capable of inhibiting K^+^ currents, not only by blocking the K^+^ current of K_V_1.2 subtype, but by altering the energetics of activation of K_V_1.1 and K_V_1.6. Moreover, experiments using six synthetic alanine point-mutated analogs further showed that a ring of basic amino acids acts as a multipoint interaction for the binding of the toxin to the channel. The AbeTx1 primary sequence is composed of 17 amino acids with a high proportion of lysines and arginines, including two disulfide bridges (Cys1–Cys4 and Cys2–Cys3), and it is devoid of aromatic or aliphatic amino acids. Secondary structure analysis reveals that AbeTx1 has a highly flexible, random-coil-like conformation, but with a tendency of structuring in the beta sheet. Its overall structure is similar to open-ended cyclic peptides found on the scorpion κ-KTx toxins family, cone snail venoms, and antimicrobial peptides.

## 1. Introduction

Voltage-gated potassium (K_V_) channels regulate diverse physiological processes, including action potential duration, neuronal excitability, and neurotransmitter release. At present, more than 50 human genes encoding 12 different families of K_V_ channels have been cloned and their structure and biochemical properties characterized. Because of the importance of mutations that are associated with pathological disorders, K_V_ channels have become an important target for developing novel therapeutic approaches and for drug design [1,2]. Since the early 1970′s, toxins have become essential tools in pharmacological and biochemical studies on K_V_ channels, and from then on, an increasing number of peptide neurotoxins that specifically target these channels have been discovered [3,4].

During the course of evolution, scorpions developed a vast array of peptide toxins to increase hunt, competition, and defense efficiency. Some of these peptides have evolved over time on the basis of common architectural motifs, displaying convergent molecular determinants and functional homologies. Scorpion toxins that target K^+^ channels (KTx) contain about 20–70 amino acids with 2–4 disulfide bridges, and they can be divided into five families: α-KTx, β-KTx, γ-KTx, κ-KTx, and ε-KTx [5,6,7,8]. κ-Hefutoxin 1, from the Asian forest black scorpion *Heterometrus fulvipes*, was the first subfamily member of the kappa-toxins (κ-KTx) active on K_V_ channels [7]. Up to date, this family is subdivided into five subfamilies and comprises more than 20 members [9]. Most of the κ-KTx toxins show a weak inhibiting activity on Shaker-type K^+^ channels, K_V_7.1, and K_V_10.1, except for κ-KTx1.3 [7,9,10,11,12,13,14]. This family of toxins adopts a unique three-dimensional fold of two parallel helices that are linked by two disulfide bridges (cystine-stabilized α/α motif) without any β-sheets. Moreover, some of these peptides share a conserved functional core that is composed of a lysine associated with a 6.6 ± 1 Å distant tyrosine responsible for their binding to K_V_ channels [7,10,11].

Sea anemone venoms are known to contain neurotoxins that are active on voltage-gated sodium (Na_V_) channels, Acid-sensitive ion channels (ASIC), and K_V_ channels [15]. The K_V_-toxins can be grouped into five structural classes according to their differences in amino acid sequences, disulfide bridge patterns, and activity profiles, and can affect channels either by blocking the current or modifying the gating mechanism. Sea anemone type 1, 2, and 5 toxins act solely as pore blocker, while type 3 toxins inhibit the currents of Shaw- and ERG-related K^+^ channels by shifting the voltage-dependence of activation in the positive direction. Type 4 toxins were indirectly assayed by competitive inhibition of the binding of ^125^I-α-dendrotoxin [15,16].

Here, we report the biochemical purification and electrophysiological characterization of the first member of a novel family (Type 6) of sea anemone K_V_-toxins, from the venom of the sea anemone *Actinia bermudensis*. AbeTx1 is a peptide with 17 amino acids and a disulfide bridge connectivity (Cys1–Cys4 and Cys2–Cys3) that is similar to that of scorpion kappa-toxins (κ-KTx), cone snail toxins and antimicrobial peptides. It is the shortest sea anemone toxin reported so far, and to the best of our knowledge, the first sea anemone K_V_-toxin that has a dual effect on Shaker-type K^+^ channels activation kinetics. Furthermore, alanine analogs were used to investigate the contribution of AbeTx1 amino acids to the molecular mechanism that is involved with K_V_ channels recognition.

## 2. Results

### 2.1. Biochemical Properties

**Figure 1:**
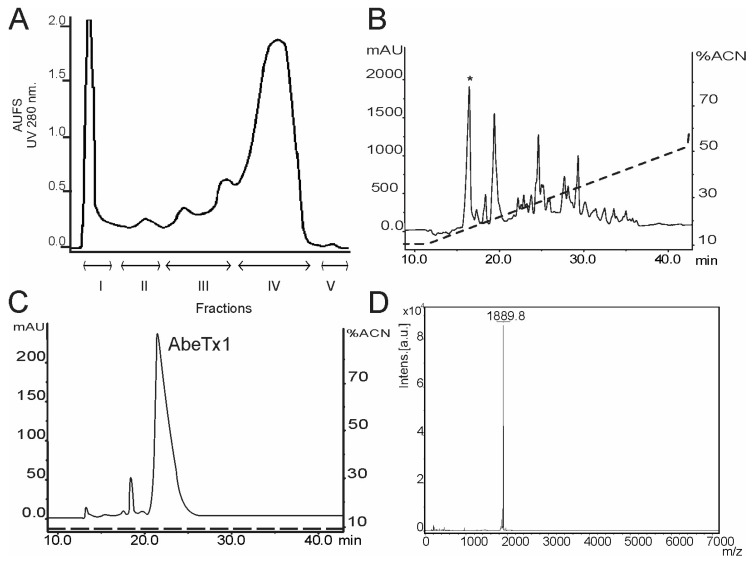
Purification and mass spectrometry characterization of AbeTx1. (**A**) Gel-filtration chromatography of *A. bermudensis* venom. Approximately 3.0 g of venom was injected into a Sephadex G-50 column, and fractions were eluted with 0.1 M ammonium acetate buffer (pH 7.0) and collected during UV (280 nm) monitoring. (**B**) Reversed-phase HPLC chromatogram of fraction III from gel filtration on a Sephadex G-50 column. The goal toxin is shown with an asterisk. (**C**) Pure AbeTx1 was obtained after a second reversed-phase HPLC step using isocratic conditions (9% solution B). (**D**) MALDI-TOF mass spectra of the HPLC-purified AbeTx1 (*m*/*z* of 1889.80 Da).

*A. bermudensis* venom was fractionated on a Sephadex G-50 column. The eluted Fraction III (Figure 1A) was then subjected to a reversed-phase HPLC chromatography, while using a linear gradient from 10 to 50% of solution B (Figure 1B). AbeTx1 toxin was separated from minor contaminants after a second rp-HPLC step, under an isocratic condition (9% of solution B) (Figure 1C). AbeTx1 purity was determined by MALDI-TOF analysis. A mass peak with an apex mass of 1889.8 Daltons was measured (Figure 1D). This experimental mass is in agreement with the calculated theoretical monoisotopic mass of 1888.875 Da. AbeTx1 amino acids could be entirely sequenced by automated Edman degradation—RCKTCSKGRCRPKPNCG—without the need for any pre-treatment of the sample. The protein sequence data reported herein will appear in the UniProt Knowledgebase (UniProtKB) under accession number C0HJD9.

**Figure 2:**
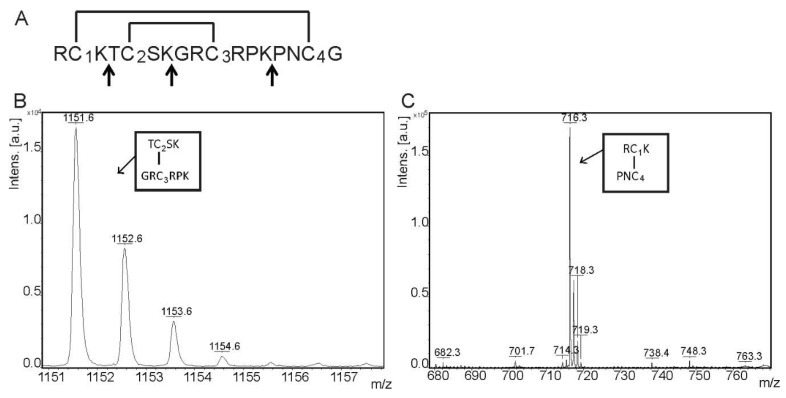
Determination of AbeTx1 cysteine framework. (**A**) Amino acid sequence obtained by Edman degradation. Arrows indicate Endopeptidase LysC cleavage sites. Above the sequence, the disulfide bridge pattern is drawn. Cysteines are indicated by numbers (1–4) (**B**,**C**) MALDI–TOF mass spectra of the digested AbeTx1. Arrows highlight molecular weights and the corresponding fragments are shown. Monoisotopic molecular weights were compared with the calculated molecular weights.

The presence of four cysteines in AbeTx1 sequence suggests the existence of two disulfide bridges. Three disulfide connectivity patterns may be inferred and they can be distinguished by cleaving AbeTx1 with Endopeptidase LysC, which hydrolyzes the peptide bonds at the carboxyl side of lysyl amino acids (Figure 2A). The molecular masses of the digested fragments were detected as single charged ions by MALDI-TOF mass spectrometry. Fragment ions were observed at *m*/*z* 171.7, 211.8, 378.9, 483.0, 568.0, 716.3, 871.4, 1084.5, 1151.6, 1216.6, 1429.8, and 1712.8 Daltons (data not shown). The ion at *m*/*z* 1151.6 correspond to the digested fragment (TCSKGRCRPK) that contain the second and third cysteines (Figure 2B), and an ion at *m*/*z* 716.3 correspond to the fragment peptide (RCKPNC) comprising the first and the fourth cysteines (Figure 2C). The other mass signals that were observed represented intermediate products of digestion.

**Figure 3:**
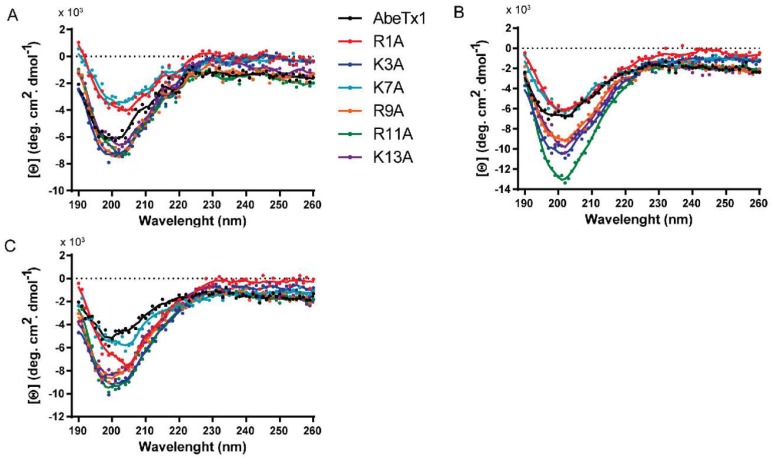
Circular dichroism (CD) spectra of AbeTx1 and six alanine point-mutated analogs in different environmental conditions. (**A**) CD buffer, (**B**) CD buffer containing POPC (100 mol%) Large Unilamellar Vesicles (LUVs), and (**C**) CD buffer containing POPC:POPS (80:20 mol%) LUVs. The LUVs concentration was fixed at 100 μM.

Furthermore, to gain insight into the relation between AbeTx1 structure and its functional activity, we next looked for its intrinsic secondary structure. Circular dichroism spectra of AbeTx1 and six alanine point-mutated analogs displayed a characteristic negative band at ≈200 nm in CD Buffer (Figure 3A), and in the presence of large unilamellar vesicles (LUVs) consisting of zwitterionic phospholipid (POPC) (Figure 3B) or a mixed phospholipid composition (POPC:POPS) with a negative net charge (Figure 3C). Albeit the similarity of all the spectra shapes, we observed differences in the spectra intensity, which may indicate different binding affinities.

The deconvolution of CD spectra (Table 1) shows a high content of random coil-like conformations, but with a tendency of structuring in beta-sheet. To our knowledge, AbeTx1 structural features are novel within the already described families of sea anemone K_V_-channel toxins.

**Figure 4:**
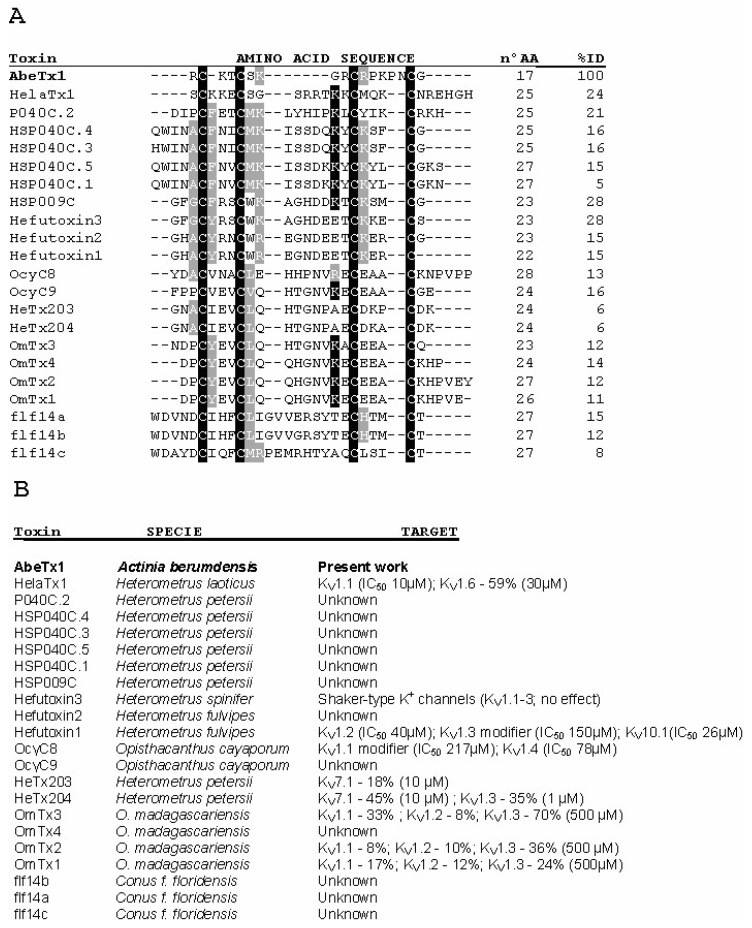
Multiple sequence alignment of AbeTx1 with scorpion κ-KTx family members and conotoxins. (**A**) The alignment is based on the cysteines (dark background). Amino acid identities (black boxes) and similarities (grey boxes) are shown. %ID is the identity score of the peptide compared to AbeTx1. (**B**) Species names and biological targets (IC_50_ values or % of current inhibition) are depicted.

The AbeTx1 amino acid sequence was used to carry out a similarities search while using BLAST [17]. Interestingly, our search did not retrieve any significant sequence similarity to the reported peptide. However, voltage-gated potassium channel toxins from scorpions and *Conus* venoms were found to have a similar cysteine pattern (Figure 4) to that of AbeTx1. All of these peptides contained four cysteines that were separated by three loops of variable size, and they share a common three-dimensional Cs α/α motif (helix-loop-helix fold), where the two α-helices are linked by the two disulfide bridges [7,9,10,11,12,18,19].

### 2.2. Pharmacological Profile

**Figure 5:**
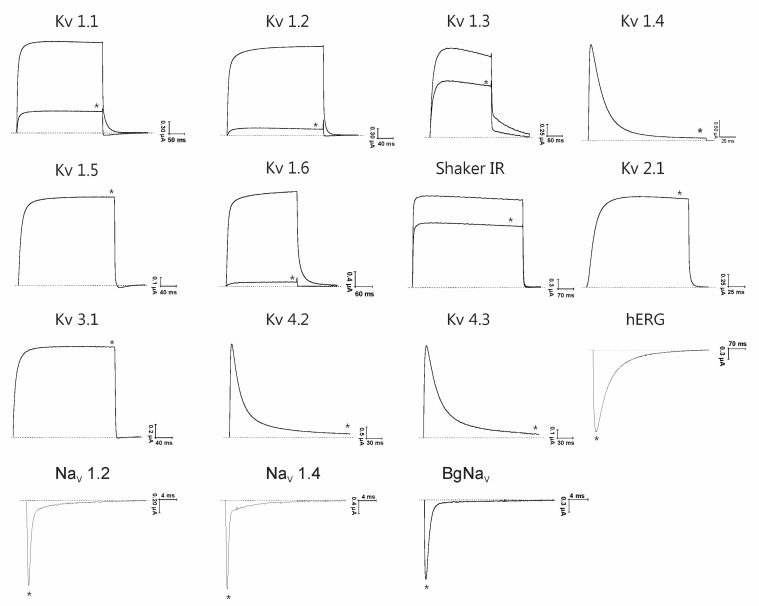
Activity of AbeTx1 on K_V_ channel subtypes of different subfamilies and three Na_V_ channels isoforms. Representative traces under control and after application of 3 µM of AbeTx1 are shown. The asterisk indicates steady-state current traces after toxin application. The dotted line indicates the zero-current level.

AbeTx1 was subjected to a screening on a wide range of 15 ion channels. Its activity was investigated on 12 voltage-gated potassium channels (K_V_1.1–K_V_1.6, K_V_2.1, K_V_3.1, K_V_4.2, K_V_4.3, Shaker IR, and hERG) and three voltage-gated sodium channels (Na_V_1.2, Na_V_1.4, and the insect channel BgNa_V_1.1). AbeTx1 showed no activity on Na_V_ channels at 3 µM concentration (Figure 5). Interestingly, the same concentration could inhibit the current of K_V_1.1 (83.67 ± 1.45%), K_V_1.2 (95.33 ± 1.45%), K_V_1.3 (19.67 ± 2.18%), K_V_1.6 (95.00 ± 2.44%), and Shaker IR (15.50 ± 1.80%) subtypes, while the other K_V_ channel isoforms from the Shaker (K_V_1.4 and K_V_1.5), Shab (K_V_2.1), Shaw (K_V_3.1), Shal (K_V_4.2-4.3), and Erg (K_V_11.1) subfamilies are not affected (Figure 5).

**Figure 6:**
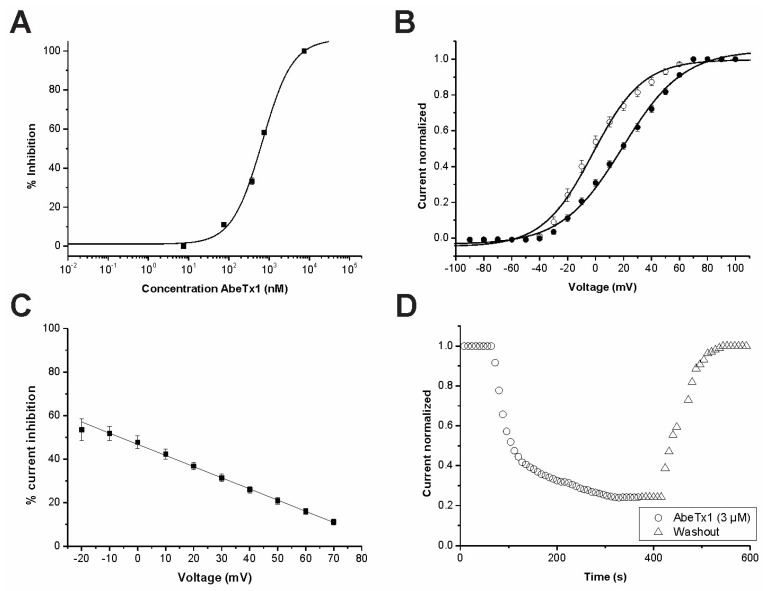
Electrophysiological characterization of AbeTx1 on K_V_1.1 isoform. (**A**) Concentration-response curve. The curve was obtained by plotting the percentage blocked current as a function of increasing toxin concentrations, fitted with the Hill equation (solid line). (**B**) Current-voltage relationships in control condition and in the presence of AbeTx1 (600 nM). Current traces were evoked by 10 mV depolarization steps from a holding potential of −90 mV. Open circles indicate the control condition; closed circles indicate the addition of toxin. The solid lines represent the average Boltzmann fits in control conditions and in presence of AbeTx1 (600 nM). (**C**) Percentage of currents inhibition after application of AbeTx1 (600 nM). The percentage of inhibition was calculated according to the formula (1 − I_Toxin_/I_Control_) × 100, where the peak currents in the presence and absence of AbeTx1 (600 nM) were measured by depolarization to the indicated voltages from a holding potential of −90 mV. Fit line is from a linear function. (**D**) Representative experiment of the time course current inhibition with AbeTx1 (3 µM) and the reversibility hereof. Control (circles), washout (triangle symbols). Plots shown are a representative of at least three individual experiments.

**Figure 7:**
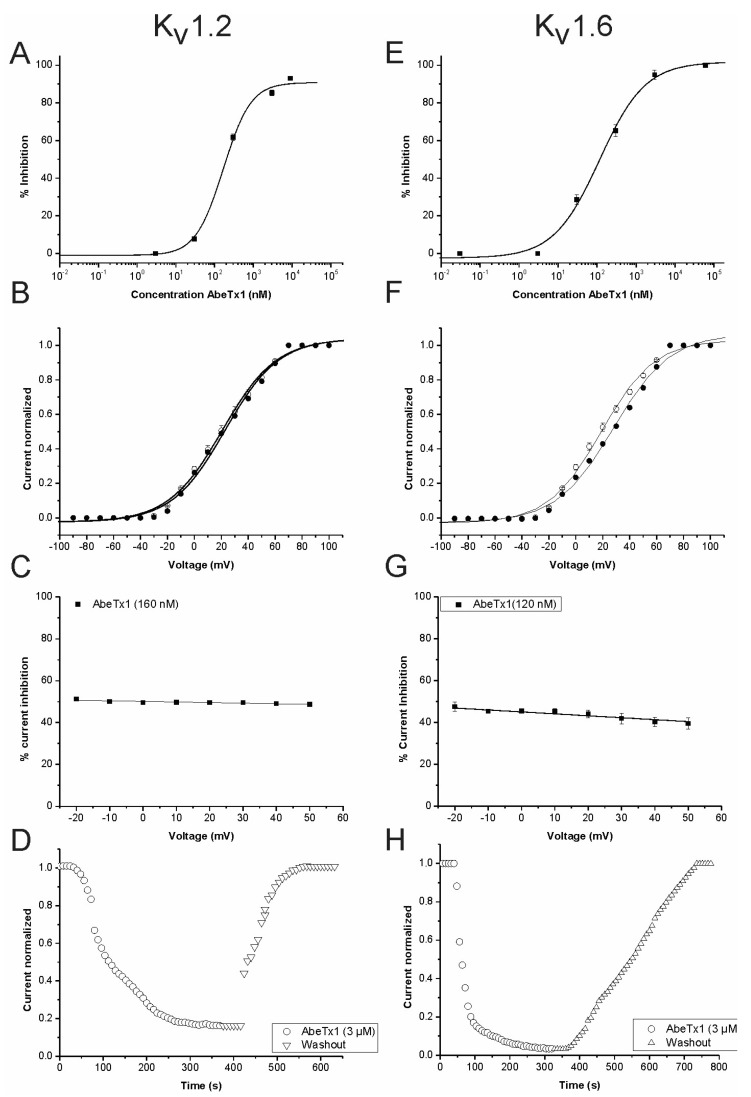
Electrophysiological experiments of AbeTx1 on K_V_1.2 and K_V_1.6 channels. (**A**,**E**) Concentration-response curve. The curve was obtained by plotting the percentage blocked current as a function of increasing toxin concentrations, fitted with the Hill equation (solid line). (**B**,**F**) Current-voltage relationships in control condition and in the presence of AbeTx1. Current traces were evoked by 10 mV depolarization steps from a holding potential of −90 mV. Open circles indicate the control condition; closed circles indicate the addition of toxin. The solid lines represent the average Boltzmann fits in control conditions and in presence of AbeTx1. (**C**,**G**) Percentage of current inhibition after the application of AbeTx1. The percentage of inhibition was calculated according to the formula. (1 − I_Toxin_/I_Control_) × 100, where the peak currents in the presence and absence of toxin were measured by depolarization to the indicated voltages from a holding potential of −90 mV. Fit line is from a linear function. (**D**,**H**) Representative experiment of the time course current inhibition with AbeTx1 (3 µM) and the reversibility hereof. Control (circles), washout (triangle symbols). Plots shown are a representative of at least three individual experiments.

In order to characterize the potency and selectivity profile, concentration-response curves were constructed for AbeTx1. IC_50_ values yielded 671.95 ± 150.31 nanomolar (nM) for K_V_1.1 (Figure 6A), 167.36 ± 38.58 for K_V_1.2 (Figure 7A), and 115.68 ± 31.44 nM for K_V_1.6 (Figure 7E). To elucidate whether AbeTx1 inhibited the current through a physical obstruction of the K_V_1.1 channel pore or act as a gating modifier, current-voltage (I–V) experiments were performed. The currents were inhibited at the test potentials from −90 to 100 mV. The I–V curve in the control experiments and in the presence of native toxin was characterized by a voltage for half-maximal activation (V_1/2_) of −1.94 ± 1.34 mV and 17.88 ± 1.26 mV, respectively (Figure 6B). The V_1/2_ of activation of K_V_1.1 is considered to be statistically significant (*p* < 0.0001). This shows that the inhibition of K_V_1.1 channels in the presence of AbeTx1 (600 nM) is associated with a depolarizing shift in the voltage-dependence of channel opening (n = 6). Moreover, the toxin-induced inhibition of K_V_1.1 channels is voltage dependent since differences in the degree of block could be observed in a range of test potentials (n = 6). At higher membrane potentials, the reduction of the K^+^ current by the toxin was decreased from 53.47 ± 5.07% at –20 mV to 11.07 ± 1.12% at +70 mV (Figure 6C). Washout experiments allow for us to determine that AbeTx1 is presumably binding the outer vestibule of the channel since toxin effect is reversible and current is completely recovered (Figure 6D).

We also investigated the AbeTx1 mechanism of action on K_V_1.2 and K_V_1.6 channel isoforms. Current-voltage (I–V) experiments were performed at the test potentials from −90 to 100 mV and the inhibition of K_V_1.2 was not associated with a change in the shape of the I–V relationship. The K_V_1.2 control curve and the curve in the presence of AbeTx1 (160 nM) were characterized by V_1/2_ values of 20.72 ± 1.33 mV and 22.82 ± 1.55 mV, respectively (*p* = 0.3620) (Figure 7B). Moreover, the K_V_1.6 control curve and the curve in the presence of toxin (120 nM) were characterized by V_1/2_ values of 18.60 ± 1.24 mV and 26.35 ± 1.52 mV, respectively (Figure 7F). The V_1/2_ of activation of K_V_1.6 is considered to be statistically significant (*p* = 0.0168). The K^+^ current inhibition of both K_V_ isoforms by the toxin was voltage-independent, since the degree of block is not different in the range of test potentials from −20 to +50 mV (Figure 7C,G). The toxin binding on both K_V_ channels is reversible—since the current was completely recovered upon washout—indicating that the binding site is presumably the outer side of the channel (Figure 7D,H).

**Figure 8:**
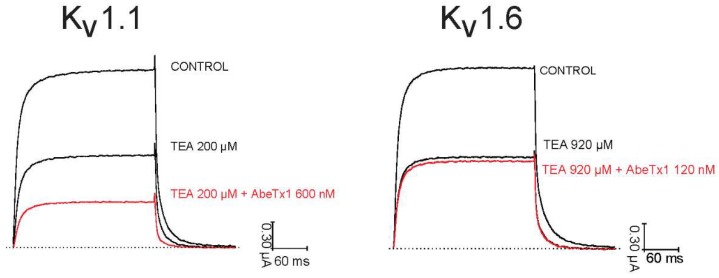
Competition between TEA_EXT_ and AbeTx1 on K_V_1.1 and K_V_1.6. Shown are the currents for control, steady-state block by TEA_EXT_ (200 µM for K_V_1.1 and 920 µM for K_V_1.6) and the subsequent effect of AbeTx1 (600 nM for K_V_1.1 and 120 nM for K_V_1.6) together with tetraethylammonium (TEA). Currents were evoked by 500 ms depolarization to 0 mV followed by a 500 ms pulse to -50 mV, from a holding potential of −90 mV.

Competitive binding experiments using tetraethylammonium (TEA) were performed on K_V_1.1 and K_V_1.6. First, 200 μM (IC_50_) TEA was applied to the bath solution and it resulted in a reduction of K_V_1.1 current to 52.20 ± 1.74%. Next, AbeTx1 (600 nM) was applied to the bath solution already containing the TEA and a reduction of 71.20 ± 2.18% was observed (n = 6), indicating that the binding sites of both ligands are not completely overlapping (Figure 8). Similar experiments were performed on K_V_1.6. After apply 920 μM (IC_50_) of TEA, K^+^ currents were reduced to 51.50 ± 0.65%. Then, when AbeTx1 (120 nM) was applied, the reduction of 55.33 ± 2.47% was observed (Figure 8).

**Figure 9:**
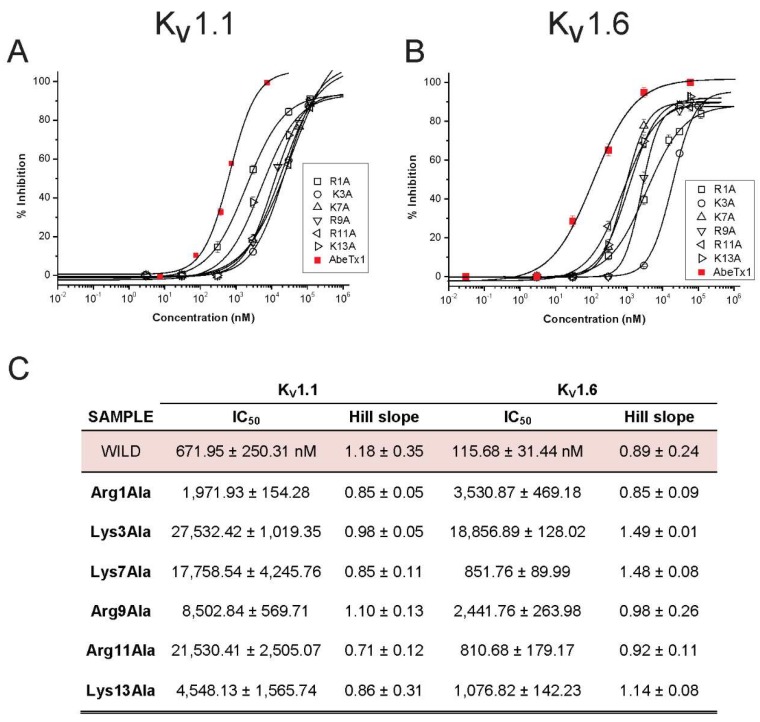
Concentration-response analysis of the effect of AbeTx1 point mutations. (**A**,**B**) Concentration-response curves on K_V_1.1 and K_V_1.6 are, respectively, shown as a plot of the percentage blocked current as a function of increasing toxin concentrations, fitted with the Hill equation (solid line). Currents were evoked by 500 ms depolarization to 0 mV followed by a 500 ms pulse to −50 mV, from a holding potential of −90 mV. (**C**) IC_50_ values and Hill coefficient were obtained by fitting the percentage blocked current as a function of increasing toxin concentrations with the Hill equation.

Furthermore, six synthetic alanine point-mutated analogs were tested on K_V_1.1 and K_V_1.6. arginine at position 1 (Arg1Ala), Lys3Ala, Lys7Ala, Arg9Ala, Arg11Ala, and Lys13Ala were substituted based on the rationale that a ring of basic amino acids present in various K_V_-acting toxins plays a pivotal role in the recognition and interaction with the K_V_ channel outer vestibule. When compared to native AbeTx1, all alanine substitutions showed a shift of the IC_50_ values to the micromolar range when tested on K_V_1.1. The three alanine analogs Lys3Ala, Lys7Ala, and Arg11Ala showed the most significant loss of activity (41, 26, and 32-fold, respectively), whereas Arg1Ala, Arg9Ala, and Lys13Ala induced a milder decrease of blocking activity (3, 6, and 12-fold, respectively). The results from concentration-response experiments and the Hill coefficient are given in Figure 9. Similar decreased blocking activity on K_V_1.6 channel was observed. Arg1Ala and Arg9Ala greatly affect AbeTx1 affinity (30 and 21-fold, respectively), and Lys3Ala resulted in the most significant decline (156-fold) of inhibition. Lys7Ala, Arg11Ala, and Lys13Ala induced a moderate reduction on toxin potency (~7 to 10-fold) when compared to wild AbeTx1 (Figure 9). Amino acid Lys-3 has been shown to be the most important amino acid for AbeTx1 binding to K_V_1.1 and K_V_1.6.

## 3. Discussion

Proteomics and transcriptomics studies have shown that peptide diversity in sea anemone venoms is more complex than previously expected, indicating that a great number of new members of known classes and novel types of toxins remains unexplored [20,21,22]. Recently, we have structurally and functionally characterized two new members of sea anemone type 1 toxins, as well as a novel family (type 5) of K_V_-toxins, purified from the venom of *B. caissarum* population from Saint Peter and Saint Paul Archipelago, Brazil [16,23]. In the present work, we have investigated the neurotoxic components of *A. bermudensis* venom, and the first representative member of a novel family of peptide toxins has been discovered. AbeTx1 was purified by gel filtration and by two reversed-phase chromatography steps (Figure 1). Homogeneity was confirmed by mass spectrometry analysis, as well as through the amino acid sequence determination (only one amino acid per cycle of the Edman degradation). AbeTx1 is a single-chain peptide containing 17 amino acids cross-linked by two disulfide bridges (Cys1–Cys4 and Cys2–Cys3). Since the primary structure of AbeTx1 has three loops—each one of them with one lysine—between the four cysteines, we decided to cleavage the peptide using Endopeptidase LysC, which hydrolyzes peptide bonds at the carboxyl side of lysyl amino acids (Figure 2). Similar to AbeTx1, other sea anemone peptides with four cysteines have been purified and functionally characterized. Sea anemone type 4 K_V_-toxins is represented by SHTX-I and -II toxins, purified from the *Stichodactyla haddoni* body extracts. These peptides have two disulfide bridges that are formed between the first cysteine and the third cysteine and the second and the fourth [24]. Moreover, by similarity, the position of the two disulfide bridges in BcgIII 23.41 [25] and U-SHTX-Sdd1 [21] (from the venom of the Brazilian sea anemones *B. cangicum* and *S. duerdeni*, respectively) was assumed to be the same. BcgIII 23.41 increased the amplitude and duration of the action potentials (a common feature for Na_V_-toxins) when being tested on crab leg sensory nerve, while U-SHTX-Sdd1 has no activity on voltage-gated potassium channels expressed in *X. laevis* oocytes. Recently, a new peptide (π-AnmTX Ugr 9a-1, from the sea anemone *Urticina grebelnyi*) adopting a unique β-hairpin motif and with a similar cysteine pattern to SHTX–I and –II, was found to inhibit the transient and the sustained current of human Acid-sensing Ion Channel 3 [26]. AbeTx1 and Ala-mutant analogs secondary structure, irrespective of varying the surface charge and hydrophobicity of the environment, shows a high content of random coil-like conformations, but with a tendency of structuring in beta-sheet (Figure 3 and Table 1). Interestingly, predictions of the AbeTx1 secondary structure while using the platforms SCRATCH Protein Predictor [27] and PROCHECK [28] also inferred the same random-coiled arrangement. Therefore, when considering the elucidation of AbeTx1 primary structure that is substantially different from the sea anemone K_V_-toxins that are described above (the highest identity received was 25%), its unique disulfide bridge pattern, secondary structure elements, and biological target, we are allowed to classify AbeTx1 as a novel family of potassium channel toxins from sea anemones, named Type 6 K_V_-toxins (Figure S1).

Kappa-toxins function was first hypothesized as a potassium channel toxin based on the presence of a ‘functional dyad’ composed of a lysine associated with a 6.6 ± 1 Å distant tyrosine, being comparable to other K_V_-toxins [7,29]. However, since the pharmacological characterization of the first scorpion κ-KTx toxin, it has been questioned if K_V_ channels are the primary target of these peptides, since IC_50_ values ranging from 10 to 217 µM and concentrations up to 500 µM are sometimes needed to block K^+^ currents (Figure 4). The present pharmacological characterization showed that AbeTx1 is active against members of the Shaker-type K^+^ channels when tested on a wide range of 15 different ion channels, including three voltage-gated sodium channel isoforms. AbeTx1 blocks K_V_1.1, K_V_1.2, K_V_1.3, K_V_1.6, and Shaker IR subtypes, but with markedly different potencies (Figure 5). The electrophysiological experiments revealed a very interesting pattern of affinities towards K_V_1.1, K_V_1.2, and K_V_1.6, and thus these three isoforms were used to further characterize the effects of the toxin. K_V_1.2 and K_V_1.6 isoforms were the most sensitive to AbeTx1 with an affinity—at nanomolar concentrations—four-fold higher than K_V_1.1 channels (Figure 6A and Figure 7A,E). Despite the absence of the ‘functional dyad’s, generally thought to be responsible for a secondary anchor point to K_V_ channels and increasing potency [30], AbeTx1 showed the highest affinity for the Shaker-type K^+^ channels when compared to other toxins with a similar folding (scorpion κ-KTx toxins and conotoxins) (Figure 4). The dyad is also not present in OmTx3 (the most potent of the four Om-toxins) because of the lack of the aromatic amino acid tyrosine [10]. However, as already reported in previous publications [9,12], there is a discrepancy between the amino acid sequence submitted to the UniProtKB database (P0C1Z4) and the reported in their publication, which has a tyrosine present at position 5, similar to the other Om-toxins. Additionally, OcyC8 does not contain the functional dyad proposed for κ-Hefutoxin 1 [7] and Om-toxins [10], but possesses the amino acid valine at the position corresponding to the tyrosine, and an arginine instead of a lysine [12].

AbeTx1 slowed the activation kinetics of K_V_1.1 and K_V_1.6 channels by shifting—to more positive voltage potentials—the energy required for channel opening (Figure 6B and Figure 7F), and also showed a voltage dependence of blockage of K_V_1.1, becoming progressively less with membrane depolarization (Figure 6C). To our knowledge, this is the first identified sea anemone toxin that is capable of modifying the activation kinetics of Shaker-Type K^+^ channels [15,31,32]. However, some scorpion κ-KTx toxins showed similar electrophysiological features. κ-Hefutoxin 1 [7] and OcyC8 [12] also slow the activation kinetics of Shaker-Type K^+^ channels (K_V_1.3 and K_V_1.1 channels, respectively), whereas HelaTx1 did not alter the activation kinetics of K_V_1.1, but it showed a voltage dependence of block. While the membrane potential becomes more positive, the channel currents blockage decreases, presumably due to the high content of basic amino acids in HelaTx1 responsible for an electrostatic repulsion between toxin and channel [9]. Interestingly, AbeTx1 also has a high content of basic amino acids (theoretical pI of 9.94 vs 10.24 of HelaTx1) and at neutral pH, it has a positive charge of + 5.8, which makes even more likely the assumption that HelaTx1, as well as AbeTx1 repulsion at higher membrane voltages, is due to their cationic surface charge (Figure 6C).

Camargos and colleagues [12] propose, based on docking simulations, Hill coefficients and selective–activity differences, that OcyC8 is not blocking the pore of the channel through a direct physical obstruction of the channel, but that one OcyC8 molecule binds to one subunit of the K^+^ channel leaving the channel pore unbarred, and then a second molecule binds to another subunit, blocking the channel pore by toxin-toxin interactions. Our data do not directly address the question of whether K_V_1.1 blockage by AbeTx1 is direct or indirect, and it needs to be further investigated. However, the Hill coefficient of AbeTx1—wild-type and—calculated from the fitting of the concentration-effect curves (Figure 9) is consistent with inhibition by a single toxin molecule. Moreover, a competitive interaction between TEA applied externally and AbeTx1 was tested based on the rationale that, if both compounds act on the same site or by a similar mechanism, their co-application will not induce a supplementary block of the remaining current. However, if they possess different, non-overlapping binding sites, a potentiation of the blockage effect will be observed. Interestingly, an additional blockage effect was observed after the co-application of TEA and AbeTx1 on K_V_1.1, suggesting that their binding sites are probably only partially overlapping (Figure 8). Since TEA inhibits K_V_ channel function by binding within the ion conduction pathway, obstructing potassium flow by interacting simultaneously with the tyrosine side chains of the selectivity filter on all four subunits [33,34], we might suggest that AbeTx1 is binding to amino acids adjacent to the selectivity filter or even remotely from the K_V_1.1 channel inner pore [35,36,37,38]. These findings are in agreement with our initial hypothesis that AbeTx1 is interacting with K_V_ channels isoforms through a multi-point interaction.

On the other hand, AbeTx1 does not modulate the voltage dependence of activation gating of K_V_1.2 channel (Figure 7B). Also, it does not show a difference on the percentage-induced block in a wide voltage range on both K_V_1.2 and K_V_1.6 isoforms (Figure 7C,G), and it blocks K^+^ currents by binding in a voltage-insensitive manner to the external vestibule (Figure 7D,H), most likely physically occluding the pore of the channel [39,40]. Therefore, the extensive pharmacological characterization presented herein (Figure 6, Figure 7, Figure 8 and Figure 9) lead us to conclude that the shift induced by AbeTx1 in the depolarizing direction of the steady-state activation curve cannot by itself account for the decrease in current seen at the tested potentials. In other words, an additional and different mechanism from gating modification alone should be invoked to explain the observed inhibition. Thus, it can be surmised that this additional mechanism is a physical pore block.

As a general line of evidence that is obtained experimentally by selected point mutations, K_V_-channel toxins are known to interact through a mechanism of action based on a multipoint interaction [29,41]. Some well-studied toxins have a ring of basic amino acids that play a role in defining K_V_ channel selectivity and affinity [42,43,44]. As already mentioned, AbeTx1 has no aromatic or aliphatic amino acids in its primary sequence to form a ‘functional dyad’, and so, our hypothesis is that AbeTx1 interacts with K_V_1.1 and K_V_1.6 channels through a ring composed of six basic amino acids (Arg-1, Lys-3, Lys-7, Arg-9, Arg-11, and Lys-13). Thus, we point-mutated each one of the AbeTx1 positively charged amino acids and is substituted by an uncharged alanine, thereby reducing the number of basic amino acids that interact with specific amino acids of the four α-subunits of the K_V_ channels, and therefore, decreasing the toxin affinity [44]. As shown in Figure 9, AbeTx1 interact through a multi-point interaction process, in which each interaction taken individually does not contribute equally to the binding strength of the peptide, and so, AbeTx1 high-affinity binding results from the summed effects of these numerous toxin-channel interactions. In particular, the substitution of Lys-3 resulted in the most significant loss of activity, suggesting that Lys-3 is a key basic amino acid that determines AbeTx1 binding to K_V_ channels. The other five mutants (Arg1Ala, Lys7Ala, Arg9Ala, Arg11Ala, and Lys13Ala) caused different and weaker effects on toxin binding. When comparing their affinity on both K_V_ isoforms tested, it seems that AbeTx1 may be docked into different orientations with respect to the K_V_1.1 and K_V_1.6 channels, in agreement with TEA binding competition experiments (Figure 8). Circular dichroism of all synthetic analogs showed similar secondary structures pattern as native (Figure 3 and Table 1), indicating that the reduced activity of these analogs is probably due to the loss of their side chains. Also, suggest that the positive electrostatic potential on the surface of AbeTx1 is likely required for higher binding affinity with K_V_ channels (net charge of wild AbeTx1 is +5.8 at neutral pH, whereas for the six mutant peptides is + 4.8). However, it should be mentioned that our results do not address whether other amino acids than those of the ring of basic amino acids might also be contributing to AbeTx1 binding affinity. For instance, Asn-30 of AgTx2 is reportedly pivotal to the stabilization of the toxin-channel complex [45], and alanine substitution of Asn-26 in Spinoxin also resulted in the significant loss of activity [46]. Sea anemone toxin BgK binding site comprises seven amino acids, including Asn-19, which induced an affinity decrease of 20-fold when substituted by an alanine [47]. AbeTx1 has an asparagine at position 15 that might contribute to the toxin multi-point interaction onto the K_V_ channel. Also, AbeTx1 has four cysteines, two glycines, and two prolines that are characteristic amino acids involved with the conformation of peptides, but might also contribute to toxin binding. Further research will provide insights into the role of these amino acids.

In conclusion, we have shown the biochemical properties of AbeTx1 as well as its selective activity toward K_V_ channels and, based on our findings, we propose to classify this toxin as the first member of a novel family of potassium channel toxins from sea anemones named sea anemone Type 6 K_V_-toxins. AbeTx1 is the shortest sea anemone K_V_-toxin and the first that is capable of blocking the current of different K_V_ channel isoforms through distinct but cooperative mechanisms of action, i.e. acting through allosteric effects as well as a pore blocker. Alanine point mutations allow for us to determine that AbeTx1 interact with K_V_ channels through a ring of six basic amino acids (Arg-1, Lys-3, Lys-7, Arg-9, Arg-11, and Lys-13) that are involved on its high affinity, although interaction through other amino acids cannot be discarded.

## 4. Materials and Methods

### 4.1. Venom Extraction and Purification of AbeTx1

Specimens of *Actinia bermudensis* were collected at São Sebastião beach, São Paulo, Brazil. The venom was obtained by electrical stimulation and fractionated as previously described [23]. Pure AbeTx1 toxin was obtained after two reversed-phase chromatography on HPLC ÄKTA Purifier system (GE Healthcare, Uppsala, Sweden), using a semi-preparative CAPCELL PAK C-18 column (1 × 25 cm; Shiseido Corp., Kyoto, Japan). Solution A was 0.1% TFA in water, solution B was acetonitrile containing 0.1% trifluoroacetic acid (TFA). First, peptide elution was achieved while using a linear gradient from 10% to 50% of solution B at a flow rate of 2.5 mL·min^−1^. Afterward, an isocratic condition of 9% of solution B at a flow rate of 1 mL·min^−1^ was performed using the same column. AbeTx1 protein content was estimated using the commercial Pierce™ BCA Protein Assay kit (Thermo Scientific, Waltham, MA, USA) according to the manufacturer’s instruction, and its homogeneity was verified by mass spectrometry analysis.

### 4.2. Mass Spectrometry Analysis

Mass spectra were acquired under linear and reflectron modes, using a Bruker Ultraflex II MALDI-TOF/ TOF controlled by FLEXCONTROL 3.0 software (Bruker Daltonics, Bremen, Germany). External calibration was performed while using Peptide Standard Calibration II (Bruker Daltonics) and Peptide Calibration Mix 3 (PepMix3; New England Biolabs, Ipswich, MA, USA) for reflection and linear modes, respectively. The matrix, α-cyano-4-hydroxycinnamic acid (Sigma-Aldrich, St. Louis, MO, USA) was prepared at a concentration of 20 mg·mL^−1^ in 1:1 acetonitrile containing 0.1% TFA solution. AbeTx1 solution (1μL) dropped onto the MALDI sample plate (384 positions; Bruker Daltonics) was added to the matrix solution (1μL) and dried at room temperature. Spectra were analyzed using thebFLEXANALYSIS 3.0 program (Bruker Daltonics Software).

### 4.3. AbeTx1 Disulfide Bonds Pattern Determination

AbeTx1 sample was digested with Endopeptidase LysC (Sigma-Aldrich) at 37 °C for 30, 60, and 120 min, in an enzyme:toxin ratio of 1:10, 1:20 and 1:100 (*w*/*w*) in Tris-HCl (150 mM, pH 8.5). Molecular weights of enzymatic fragments were acquired, as described before, using a Bruker Ultraflex II MALDI-TOF/TOF (Bruker Daltonics).

### 4.4. Amino Acid Sequence Determination

Native AbeTx1 (100 pmol) was sequenced by Edman degradation using the automated PPSQ-33A protein sequencer (Shimadzu, Kyoto, Japan) coupled to reversed-phase separation of phenylthiohydantoin (PTH)-amino acids on a WAKOSIL-PTH (4.6 × 250 mm) column (Wako, Osaka, Japan), according to the manufacturer’s instructions.

### 4.5. Large Unilamellar Vesicles (LUVs) Preparation

The lipid stock solutions in chloroform were transferred to glass tubes in the appropriate proportions at a final concentration of 5 mM and the solvent was evaporated under N_2_ flow. The samples were dried under vacuum for 3 h to remove solvent traces. The lipid films were hydrated with CD buffer (5 mM Tris and 150 mM NaF, pH 7.4, adjusted with H3BO3). The lipid suspensions were thoroughly vortex mixed and submitted to 31 extrusions through two-stacked 100 nm polycarbonate membranes using an Avanti mini-extruder (Avanti Polar Lipids, Alabaster, Alabama, USA). The phospholipid content was measured by the Rouser method [48]. The lipid films were prepared in the following compositions: (A) 100 mol% of 1-palmitoyl-2-oleoylphosphatidylcholine (POPC) and (B) 80:20 mol% of POPC: 1-palmitoyl-2-oleoylphosphatidylserine (POPS).

### 4.6. Circular Dichroism

Circular Dichroism (CD) experiments were performed in a Chirascan-plus qCD (Applied Photophysics Ltd, Leatherhead, Surrey, UK) in a 0.1 cm path length quartz cuvette. AbeTx1 and analogs (10 µM) were added in a cuvette containing CD Buffer or CD Buffer and POPC (100 mol%) or POPC: POPS (80:20 mol%) LUVs (100 µM). The CD spectra were collected from 190 to 260 nm, at 25 °C, with a bandwidth of 1 nm, step of 1 nm, time-per-point of 0.1 s, and current time-per-point of 0.1 s. Each CD spectra was averaged over eight repetitions. The observed ellipticity θ (mdeg) was converted to mean amino acid ellipticity θ (deg·cm^2^·dmol^−1^) after the baseline correction, using the relationship: [θ] = (100.θ)/(l.c.n). Where l is the path length in centimeters, c is peptide millimolar concentration and n the number of peptide amino acids. A lipid baseline spectrum was subtracted from all peptide/lipid spectra.

### 4.7. AbeTx1 Analogs Synthesis–Alanine Substitution

Six synthetic AbeTx1 analogs (1–4 mg) were purchased from GenScript Corporation (NY) with purity higher than 98% and the same disulfide bonds pattern (Cys1–Cys4 and Cys2–Cys3) as native AbeTx1. Positively charged amino acids (Arg1, Lys3, Lys7, Arg9, Arg11, and Lys13) were substituted by an uncharged alanine. Analogs are designated by three letters and number indicating the identity and position of the six substituted amino acids, followed by three letters indicating the identity of the replacement amino acid. Quality control of the six analogs was confirmed by HPLC, mass spectrometry, and Edman degradation sequencing, as described above.

### 4.8. Heterologous Expression of Ion Channels in Xenopus Laevis Oocytes

The pharmacological effect of AbeTx1 was analyzed by heterologous expression of 12 voltage-gated potassium channels isoforms (rK_V_1.1, Genbank NM173095; rK_V_1.2, Genbank NM012970; hK_V_1.3, Genbank L23499; rK_V_1.4,Genbank NM012971; rK_V_1.5, Genbank NM012972; rK_V_1.6, Genbank NM575671; Shaker IR from Drosophila melanogaster, Genbank CG12348; rK_V_2.1, Genbank NM013186; hK_V_3.1, Genbank NM004976; rK_V_4.2, Genbank NM031730; rK_V_4.3, Genbank NM031739 and hERG, Genbank NM000238), and three voltage-gated sodium channel subtypes (rNa_V_1.2, Genbank NM012647; rNa_V_1.4, Genbank M26643 and BgNa_V_1.1, from *Blattella germanica*) (r, rat; h, human) in oocytes from *Xenopus laevis*. The linearized plasmids were transcribed using a T7 or SP6 mMESSAGE-mMACHINE transcription kit (Life Technologies, Austin, TX, USA). Oocytes were injected with 20–50 nL of cRNA at a concentration of 1 ng·nL^−1^ using a microinjector (Drummond Scientific, Broomall, PA, USA). The oocytes were stored for 1–3 days at 16 °C until sufficient expression of voltage-gated ion channels was achieved. Oocytes were maintained in an ND96 solution (in mM: 96 NaCl, 2 KCl, 1.8 CaCl2, 2 MgCl2 and 5 HEPES; pH 7.4), supplemented with 50 µg·mL^−1^ gentamicin sulfate.

### 4.9. Electrophysiological Recordings

Two-electrode voltage-clamp recordings were performed at room temperature (18–22 °C) using a Geneclamp 500 amplifier (Molecular Devices, Sunnyvale, CA, USA) controlled by a pClamp data acquisition system (Axon Instruments, Union City, CA, USA). ND96 was used as bath solution. Whole-cell currents from oocytes were recorded 1–3 days after injection. Voltage and current electrodes were filled with KCl (3 M). The resistance of both electrodes was kept between 0.8–1.0 MΩ. The elicited currents were filtered at 0.5 or 2 kHz using a four-pole low-pass Bessel filter. Leak subtraction was performed using a -P/4 protocol. K_V_1.1-K_V_1.6 and Shaker IR currents were evoked by 500 ms depolarization to 0 mV, followed by a 500 ms pulse to −50 mV, from a holding potential of −90 mV. K_V_2.1, K_V_3.1, K_V_4.2, and K_V_4.3 currents were elicited by 500 ms pulses to + 20 mV from a holding potential of −90 mV. Current traces of hERG channels were elicited by applying a +40 mV pulse for 2.5 s, followed by a step to −120 mV for 2.5 s. Sodium current traces were evoked by 100 ms depolarization steps from a holding potential of −90 mV to 0 mV. Concentration-response curves were constructed by adding different AbeTx1 or analogs concentrations directly to the bath solution. The percentage of the K_V_ blockade was plotted against the logarithm of the applied concentrations and fitted with the Hill Equation (1):

y = 100/[1 + (IC_50_/[toxin]) × *h*] (1)

where y is the amplitude of the toxin-induced effect, IC_50_ is the toxin concentration at half-maximal efficacy, [toxin] is the toxin concentration, and *h* is the Hill coefficient. To investigate the effects on the voltage dependence of activation, current traces were evoked by 10 mV depolarization steps from a holding potential of −90 mV. Data were normalized to the maximal K^+^ current amplitude, plotted against prepulse potential, and fitted using the Boltzmann Equation (2):

IK/Imax = [1 + (exp(Vg − V)/k] − 1(2)

where Imax represents maximal potassium current (IK), Vg is the voltage corresponding to half-maximal current, and k is the slope factor. Each experimental data represents mean ± standard error of at least three oocytes (n ≥ 3) from different frogs. Effect of the toxin on current-voltage experiments was statistically examined by comparing the control condition and the presence of toxin using the Student’s paired *t*-test. *P*-values < 0.05 are considered to be statistically significant. Data were analyzed using Clampfit 10.4 (Molecular Devices, Sunnyvale, CA, USA) and Origin v8.1 software (Origin Lab, Northampton, MA, USA), under the institutional license of the University of São Paulo.

### 4.10. Bioinformatics

BLAST searches [17] using AbeTx1 amino acid sequence as the query sequence were performed against the data sets of GenBank and Protein Knowledgebase (UniProtKB). A multiple sequence alignment of AbeTx1, neurotoxins from marine cone snails, and kappa-toxin subfamily members was done with ClustalW2 [49]. Sequences analyzed were that of Hefutoxin 1 and 2 (Swiss-Prot P82850 and P82851, respectively–from *Heterometrus fulvipes*) [7]; Hefutoxin 3 (Swiss-Prot P83655–*H. spinifer*) [11]; HelaTx1 (Swiss-Prot P0DJ41–*H. laoticus*) [9]; OmTx1–4 (Swiss-Prot P0C1Z3 and P0C1Z4, respectively–*Opisthacanthus madagascariensis*) [10]; OcyC8 (Swiss-Prot P86110) and OcyC9 (Swiss-Prot C5J893) (*O. cayaporum*) [12]; HSP009C, HSP053C.1 (HeTx203), HSP053C.2 (HeTx204), HSP040C.1, HSP040C.3, HSP040C.4, HSP040C.5 and HSP040C.2 (Swiss-Prot P0DJ33–P0DJ40, respectively*—H. petersii*) [13,18]; and, flf14a–c (Swiss-Prot P84705–P84707, respectively—*Conus floridanus floridensis*) [19].

## Figures and Tables

**Table 1:** Secondary structure analysis of AbeTx1 and six synthetic alanine point mutated analogs fitted by the CD spectra deconvolution software CDNN 2.1.

		WT	Arg1Ala	Lys3Ala	Lys7Ala	Arg9Ala	Arg11Ala	Lys13Ala
**Tris Buffer**	Helix	14.54	13.38	13.84	14.12	13.08	13.48	13.73
	Beta	34.75	34.96	35.83	34.6	35.67	36.03	36.18
	Turn	17.54	17.11	17.46	17.26	17.19	17.4	17.52
	Random Coil	33.17	34.55	32.87	34.1	34.06	33.09	32.65
**POPC**	Helix	14.7	13.11	14.03	13.71	13.5	13.54	13.46
	Beta	34.09	34.92	34.98	34.29	34.88	35.4	35.97
	Turn	17.38	16.98	17.41	17.06	17.15	17.29	17.37
	Random Coil	33.92	34.92	33.58	34.94	34.47	33.77	33.20
**POPC:POPS (80:20)**	Helix	13.03	13.16	13.63	12.4	13.61	13.71	13.55
	Beta	35.32	35.27	36.37	35.39	35.98	35.88	36.62
	Turn	17.06	17.11	17.57	16.82	17.46	17.49	17.57
	Random Coil	34.59	34.46	32.43	35.39	32.87	32.92	32.27

## Supplementary Materials

The following are available online at http://www.mdpi.com/1660-3397/16/10/360/s1, Figure S1: Multiple sequence alignment of sea anemone K_V_ toxins.
