# Supplementary material for: AbeTx1 Is a Novel Sea Anemone Toxin with a Dual Mechanism of Action on Shaker-Type K+ Channels Activation
Source: Mar Drugs. 2018 Oct 1;16(10):360. doi: 10.3390/md16100360 (PMC6213216; doi:10.3390/md16100360)
Supplement: Supplementary file 1 [file marinedrugs-16-00360-s001.pdf]

marine drugs

Supplementary Materials

AbeTx1 Is a Novel Sea Anemone Toxin with a Dual Mechanism of Action on Shaker-Type K^+ Channels Activation

Diego J. B. Orts [1], Steve Peigneur [2], Laíz Costa Silva-Gonçalves [3], Manoel Arcisio-Miranda [3], José Eduardo P. W. Bicudo [1] and Jan Tytgat [2,*]

1 Department of Physiology, Institute of Biosciences, University of São Paulo, 05508-090 São Paulo, Brazil; diego.orts@usp.br (D.J.B.O.); jebicudo@usp.br (J.E.P.W.B.)

2 Toxicology and Pharmacology, University of Leuven (KU Leuven), Campus Gasthuisberg O&N2, Herestraat 49, P.O. Box 922, 3000 Leuven, Belgium; steve.peigneur@pharm.kuleuven.be

3 Laboratório de Neurobiologia Estrutural e Funcional (LaNEF), Departamento de Biofísica, Universidade Federal de São Paulo, 04023-062 São Paulo, Brazil; laiz.lcs@gmail.com (L.d.C.S.-G.); arcisio.miranda@unifesp.br (M.A.-M.)

* Correspondence: jan.tytgat@pharm.kuleuven.be; Tel.: +32-16-32-34-03; Fax: +32-16-32-34-05

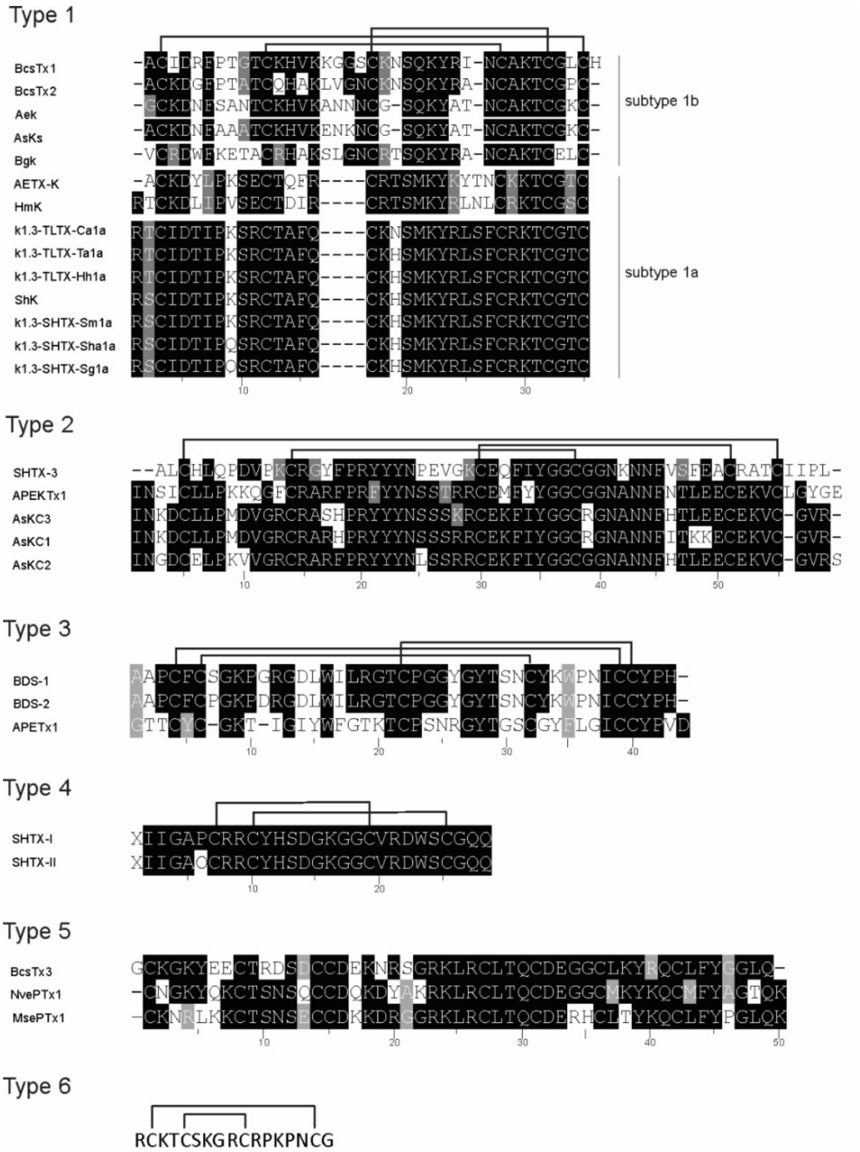

Figure S1. Multiple sequence alignment of sea anemone Kv-toxins. The amino acid sequences are aligned according to their cysteine residues. Disulfide bridge patterns are indicated. Amino acid identities (black boxes) and similarities (gray boxes) are shown. Sea anemone Type 1 Kv-toxins are BcsTx1 (Swiss-Prot C0HJC2), BcsTx2 (Swiss-Prot C0HJC3) from the venom of the *B. caissarum* population from Saint Peter and Saint Paul Archipelago, Aek (=Swiss-Prot # P81897) from *Actinia equina*, AsKs (=Swiss-Prot # Q9TWG1), from *Anemonia sulcata*, Bgk (=Swiss-Prot #P29186) from *Bunodosoma granulifera*, AETX-K (=Swiss-Prot #Q0EAE5) from *Anemonia erythraea*, HmK (=Swiss-Prot #O16846) from *Radianthus magnifica*, κ1.3-TLTX-Ca1a (=GenBank #AB595207) (*Cryptodendrum adhaesivum*), κ1.3-TLTX-Ta1a (=GenBank # AB595209) (*Thalassianthus aster*), κ1.3-TLTX-Hh1a (=GenBank #AB595208) (*Heterodactyla hemprichi*), ShK (=Swiss-Prot #P29187) (*Stichodactyla helianthus*), κ1.3-SHTX-Sm1a (=GenBank #AB595206) (*Stichodactyla mertensii*), κ1.3-SHTX-Sha1a (=GenBank #AB595205) (*Stichodactyla haddoni*), and κ1.3-SHTX-Sg1a (=GenBank #AB595204) (*Stichodactyla gigantea*). Type 2 Kv-toxins are SHTX-3 (Swiss-Prot B1B518) from *Stichodactyla haddoni*; APEKTx1 (Swiss-Prot P86862) from *A. elegantissima*, AsKC3 (Swiss-Prot Q9TWF8), AsKC1 (Swiss-Prot Q9TWG0), and AsKC2 (Swiss-Prot Q9TWF9) from *A. viridis*; Type 3 Kv-toxins are BDS-1 (Swiss-Prot P11494), BDS-2 (Swiss-Prot P59084) from *A. viridis*, and APETx1 (Swiss-Prot P61541) from *A. elegantissima*. Type 4 Kv-toxins are SHTX-I/II (Swiss-Prot P0C7W7) from *S. haddoni*. Type 5 Kv-toxins are BcsTx3 (Swiss-Prot C0HJC4) and mature portions of the putative proteins NvePTx1 (GenBank EDO47375.1) from *N. vectensis*, and MsePTx1 (GenBank FC839755.1) from *M. senile*. The newly discovered Type 6 Kv-toxin is represented by AbeTx1 from *Actinia bermudensis*.
